# Exploring the complexities of poultry respiratory microbiota: colonization, composition, and impact on health

**DOI:** 10.1186/s42523-024-00308-5

**Published:** 2024-05-06

**Authors:** Samson Oladokun, Shayan Sharif

**Affiliations:** Department of Pathobiology, Ontario Veterinary College, University of Guelph, Guelph, ON N1G 2W1 Canada

**Keywords:** Microbiota, Respiratory system, Diversity, Health, Pathology, Poultry

## Abstract

An accurate understanding of the ecology and complexity of the poultry respiratory microbiota is of utmost importance for elucidating the roles of commensal or pathogenic microorganisms in the respiratory tract, as well as their associations with health or disease outcomes in poultry. This comprehensive review delves into the intricate aspects of the poultry respiratory microbiota, focusing on its colonization patterns, composition, and impact on poultry health. Firstly, an updated overview of the current knowledge concerning the composition of the microbiota in the respiratory tract of poultry is provided, as well as the factors that influence the dynamics of community structure and diversity. Additionally, the significant role that the poultry respiratory microbiota plays in economically relevant respiratory pathobiologies that affect poultry is explored. In addition, the challenges encountered when studying the poultry respiratory microbiota are addressed, including the dynamic nature of microbial communities, site-specific variations, the need for standardized protocols, the appropriate sequencing technologies, and the limitations associated with sampling methodology. Furthermore, emerging evidence that suggests bidirectional communication between the gut and respiratory microbiota in poultry is described, where disturbances in one microbiota can impact the other. Understanding this intricate cross talk holds the potential to provide valuable insights for enhancing poultry health and disease control. It becomes evident that gaining a comprehensive understanding of the multifaceted roles of the poultry respiratory microbiota, as presented in this review, is crucial for optimizing poultry health management and improving overall outcomes in poultry production.

## Introduction

Recent projections suggest poultry will be the most consumed animal protein globally in this decade [1]. It is apparent that the fulfillment of this projection is contingent on maintaining optimum growth performance and health in poultry. Respiratory infections and diseases in poultry are reportedly the primary cause of economic losses in the poultry industry [2–4]. Emerging results indicate that the microbial community present in the avian respiratory tract plays a critical role in maintaining optimal respiratory health [4, 5].

To fulfill the primary function of gaseous exchange (oxygen and carbon dioxide) between the atmosphere and systemic circulation, the avian respiratory system anatomically consists of the nares and nasal cavities, oropharyngeal region, the larynx, which allows selective passage of air into the trachea, bronchi connected to the lungs, and their associated air sacs. The respiratory tract was traditionally thought to be sterile due to the limitations of traditional culture-based microbiology techniques [6–8]. However, the emergence of culture-independent methods like the next-generation sequencing technology have now provided proof that the avian respiratory tract plays host to a dynamic community of microorganisms, especially bacteria [5, 9, 10]. The resident microbiota of the respiratory tract plays several pathophysiological roles related to pathogen exclusion and enhancing immunocompetence [11–13]. As a result of the role of the respiratory microbiota in poultry performance and health, the study of the respiratory microbiota is thus an emerging field of research. Nevertheless, compared to the respiratory microbiota, greater focus has been placed studying the composition and metagenomic profile of the poultry intestinal microbiota [14, 15].

While a few studies have provided baseline information on the poultry respiratory microbiota [9, 13], it is important to synthesize available information into clear concepts to provide direction on the role of respiratory tract commensals and pathogens, as well as well as their relationships to health or disease outcomes in poultry. Accordingly, this review seeks to provide a comprehensive overview on poultry respiratory tract microbiota community structure, factors influencing the community structure, challenges faced in studying this microbiota, and its impact on host health and pathology.

### Poultry respiratory microbiota: pioneer colonizers and community structure

Similar to mammals, the Upper Respiratory Tract (URT) of birds comprises the oral and nasal cavities which are in continuous communication, a larynx upheld by cartilaginous plates, and the trachea. Conversely, the lower respiratory tract (LRT) comprises the syrinx, air sacs, bronchioles, and lungs [16]. Contrary to the prevailing microbial sterility dogma, there is now evidence for microbial colonization of the avian respiratory tracts [17, 18]. In view of this knowledge, the question of the origin of pioneer colonizers of the respiratory tract arises. Microbial colonization of the respiratory tract is a complex process influenced by host-related intrinsic factors and environmental extrinsic factors. Nonetheless, the pioneer colonizers of the poultry respiratory tract are hypothesized to be maternal-dependent. This hypothesis has been demonstrated in humans via a metagenomic neonatal study that showed differences in infant-mother gut microbiota resemblance based on delivery method [19]. Gantois et al. [20] exposition on the pathogenesis of *Salmonella* contamination in poultry support this hypothesis in poultry. It is reported that microbial seeding could occur via horizontal transmission during or after oviposition in the hen or vertically through the pores on eggshell membranes or eggshells. An association between maternal oviduct microbiota, yolk microbiota, and hatched chick microbiota has also been confirmed [21, 22]. Notwithstanding, well-designed longitudinal studies are needed to provide more insight on the maternal-origin hypothesis on microbial pioneer colonization in poultry, especially taking into cognizance modern poultry production systems that limit maternal hen-egg contact.

**Fig. 1:**
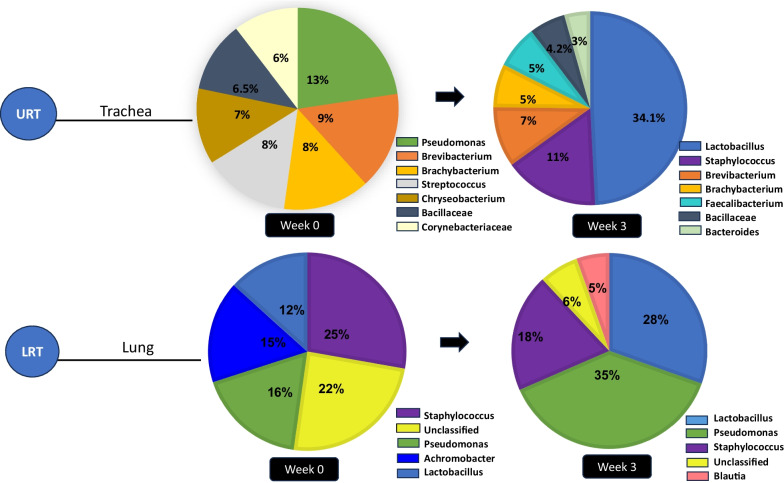
Dynamics of microbiota colonization and succession in the avian trachea and lungs. At hatch, the avian trachea (representative of the upper respiratory tract-URT) was colonized predominantly by *Pseudomonas* (13%), *Brevibacterium* (9%), *Brachybacterium* (8%), *Streptococcus* (8%), *Chryseobacterium* (7%), *Bacillaceae* (6.5%), and *Corynebacteriaceae* (6%). By week 3, the tracheal microbiota shifted, with reductions in *Brevibacterium* (6%), *Bacillaceae* (4.2%), and *Brachybacterium* (5%). Genus *Lactobacillus* emerged dominantly, constituting 34.1% of the trachea. In contrast, the lung microbiota (representative of the lower respiratory tract-LRT) displayed distinct colonization. Initially, *Staphylococcus* (25%), *Pseudomonas* (16%), *Achromobacter* (15%), and *Lactobacillus* (12%) were predominant at hatch. By week 3, the composition shifted to *Pseudomonas* (35%), *Lactobacillus* (28%), and *Staphylococcus* (18%) as the dominant genera in the avian lungs. Adapted from Glendinning et al. (2017) and Mulholland et al. (2021)

While Nehme et al. [23] made the first attempt to enumerate poultry respiratory bacterial communities, the findings of this study have limited practical use as a result of the limitations of the culture-dependent method that was employed. The study by Mulholland and colleagues [17] was the first study to adopt 16S rRNA amplicon sequencing methodology to provide a bacteria census of the poultry respiratory tract, specifically of the trachea at hatch. Data from this study affords the opportunity to speculate on the pioneer colonizers of the poultry respiratory tract. Accordingly, Fig. 1 presents information on the pioneer genera of the avian trachea. The avian trachea is seen to be predominantly (62%) dominated by the genera *Pseudomonas, Brevibacterium, Streptococcus, Chryseobacterium, Bacillaceae*, and *Corynebacteriaceae* at hatch. Using two- and seven-day-old chickens, Glendinning et al. [24] and Ngunjiri et al. [25] have also reported that genera *Pseudomonas, Staphylococcus, Actinobacillus*, and *Lactobacillus* species (*L. reuteri, L. gasseri, and L. crispatus*) are initial colonizers of the chicken nasal, tracheal, and Broncho-Alveolar Lavage (BAL) samples. At the phylum taxa, Mulholland et al. [17] report that the avian trachea is majorly colonized by four phyla at hatch, specifically Gram-positive bacteria Firmicutes (35%) and Actinobacteria (29%), Gram-negative bacteria (Proteobacteria (19%), and Bacteroidetes (9%). Although with varying order of abundance, the same bacterial phyla have been reported as pioneer colonizers of the avian gut [26, 27]. While the effects of the hatchery environment and contaminants on potential colonizers of the respiratory tract should be acknowledged, these results suggest that maternal influence plays a significant role in shaping the pioneer microbial community of the avian respiratory tract.

In terms of community structure, phyla Firmicutes, Proteobacteria, and Bacteroidetes—and to a lesser extent Actinobacteria and Tenericutes—are consistently reported as the major residents of the poultry respiratory tract [12, 25, 28]. Phyla Firmicutes and Bacteroidetes have also been reported as the predominant bacteria in the human colon [29], suggesting an evolutionary trend. The Firmicutes phylum comprises more than 200 genera, including important probiotics like *Lactobacillus, Bacillus,* and *Ruminicoccus*. Conversely, the predominant genera in the phylum Bacteroidetes, including *Bacteroides* and *Prevotella,* are known pathobionts [30]. Of note, high Firmicutes to Bacteroidetes ratios are associated with increased inflammation, fat deposition and obesity in humans [31]. A reduction in the abundance of members of the Firmicutes phylum has also been linked with several pathologies in other animals including pigs, cattle, and horses [32]. At lower taxa, as many as 144 bacterial genera have been identified in the respiratory tract of turkeys including 25 shared genera between turkeys and chickens [9, 33], emphasizing the complexity of the poultry respiratory microbiota. A detailed summary of studies presenting the predominant genera in the poultry respiratory tract is presented in Table 1. The trachea and BAL samples are the most studied sections of the URT and LRT in poultry, respectively. The avian respiratory tract comprises more facultative anaerobes than obligate anaerobes, with genera *Lactobacillus* and *Staphylococcus* being ubiquitous residents of both the URT and LRT [17, 33]. Although the genus *Lactobacillus* predominates the poultry respiratory tract [34], its relative abundance varies depending on which part of the respiratory tract is being examined. The *Lactobacillus* genus is known to comprise gram-positive facultative anaerobic bacteria that degrade polysaccharides to yield lactic acid [35]. *Lactobacillus* species are often used in the poultry industry as probiotics as a result of their beneficial properties [36]. Notwithstanding, Johnson et al. [34] have reported a negative correlation for increased *Lactobacillus* abundance in the cecum and ileum with weight of birds. This result emphasizes the need to be considerate of the type of *Lactobacillus* species, rather than the genus, when formulating probiotics applications. Common *Lactobacillus* species in the poultry respiratory tract include *Lactobacillus salivarius*, *Lactobacillus crispatus/acidophilus/gallinarum*, *Lactobacillus johnsonii/gasseri*, *Lactobacillus aviarius*, and *Lactobacillus reuteri* [34]. Depending on the metabolic property and oxygen requirement of the bacteria, they can be classified as transient or colonizing species. For example, *Lactobacillus aviaries* is known as a transient species in the trachea because it is an obligate anaerobe found in relatively lower abundance [34, 37]. Experimental studies have demonstrated that *Lactobacillus* species exhibit immunostimulatory and antiviral properties [38–40]. Additionally, *Lactobacillus acidophilus*, *Bifidobacterium bifidum*, and *Streptococcus faecalis* species have been found to induce natural antibodies against various foreign antigens in chickens [41].

**Table 1:** A comprehensive summary of studies that present the most prevalent genera observed in the respiratory tract of poultry

S/N	Type of poultry bird	Study description	Sequencing technology	Respiratory site	Predominant genera	Results	References
1	Broiler chickens	Identified bacterial taxa associated with broiler performance in antibiotic-free commercial flocks	16S V4 rRNA gene sequencing	Trachea	*Lactobacillus, Corynebacterium, Enterococcus, Ruminococcus, Staphylococcus, Streptococcus,* and *Xanthomonas*	Positively correlated with birds performance: *Candidatus Arthromitus, Corynebacterium, Dietzia, Facklamia, Leucobacter, Staphylococcus*, and *Weissella* Negatively correlated with birds performance: *Avibacterium, Achromobacter, Brevibacillus, Erysipelothrix*, Gallibacterium, and* Ornithobacterium*	[34]
2	Broiler chickens	Examined the effect of heat stress and prebiotic and probiotic supplementations on broiler tracheal bacterial communities	PCR- DGGE fingerprinting technique and bTEFAP 16S rRNA pyrosequencing	Trachea	*Aerococcus, Anaerococcus, Globicatella, Lactobacillus*, Nosocomiicoccus and *Staphylococcus*	Core trachea microbiome was dominated by *Lactobacillus* No positive effects of supplementations were observed on abundance of probiotic bacteria	[12]
3	Layer and broiler chickens	Evaluated the abundance and drug resistance of selected bacteria in the trachea of chickens under intensive and free-range production systems	Plate culture	Trachea	–	Bird production system had no effect on total bacteria count, Staphylococcus aureus, and the psychrophilic bacteria Tracheal coliform count was higher in broilers compared to layers Tracheal bacterial resistance to chloramphenicol was significantly higher in broilers compared with layers	[23]
4	Commercial poultry flocks	Provided a comprehensive analysis of the ecology of the avian respiratory microbiome throughout the grow-out phase	16S V4 rRNA Amplicon Sequencing	Trachea	*Lactobacilli, Chryseobacterium, Pseudomonas, Brevibacterium*	Asides using a metagenomic approach to study the avian respiratory microbiota, dysbiosis was also detected in the avian respiratory virome of broiler chickens diagnosed with infectious laryngotracheitis virus	[17]
5	Laying hens	Evaluated differences in the microbial community across different respiratory sites in birds of different ages	16S V1-V4 rRNA Amplicon Sequencing	buccal, nasal, and BAL	Buccal and nasal swabs: high abundance of *Lactobacillus* BAL: *Gallibacterium*, *Avibacterium*, *Acinetobacter,* and *Staphylococcus*	Significant differences exist in the composition, richness, and diversity of bacterial communities in the buccal, nasal, and BAL fluid samples between chickens of different age groups	[24]
6	Commercial turkey flocks	Bacteria census of the bacteria communities in trachea of commercial turkey flocks	16S V3-V4 rRNA Amplicon Sequencing	Trachea	*Lactobacillus, Enterococcus, Escherichia-Shigella*, and* Morganella*	Respiratory pathogens like *Ornithobacterium* and Mycoplasma that could contribute to the development of respiratory infections were identified in the turkey trachea	[33]
7	White leghorn chickens	Compared chicken respiratory microbiota profile obtained using different DNA extraction kits	16S V4 rRNA Amplicon Sequencing	Choanal, nasal cavity, trachea, and BAL	Choanal swab: *Lactobacillus, Streptococcus, Vibrio,* and* Romboutsia,* Nasal swab: *Lactobacillus, Streptococcus,* and* Acinetobacter* Trachea- Lactobacillus, *Streptococcus, Escherichia-Shigella, Romboutsia, Vibrio* BAL: *Lactobacillus, Streptococcus,* and* Vibrio*	DNA extraction kit type had minimal effects on taxonomic composition and diversity	[45]
8	Layer chickens	Defined the baseline bacterial microbiota in the upper respiratory tract of commercial layer chickens	16S V4 rRNA Amplicon Sequencing	Nasal, and trachea	Nasal: *Staphylococcus, Lactobacillus, Ruminococcus, Enterococcus,* and *Deinococcus* Trachea: *Burkholderiaceae, Lactobacillus, Enterococcus,* and *Avibacterium,* and *Mycoplasma*	Sampling site, age of birds, and farm stage had dominant effects on the taxonomic composition and dynamics of core bacteria	[25]
9	Turkey	Presented a census of bacterial residents in the turkey respiratory tract	16S V4 rRNA Amplicon Sequencing	Nasal cavity and Trachea	Nasal: *Staphylococcus, Macrococcus, Rothia, Lactobacillus, Bacillaceae,* and *Deinococcus* Trachea: *Mycoplasma, Moraxella, Macrococcus,* and *Lactobacillus*	*Deinococcus* and *Ornithobacterium* were negatively correlated with body weight	[18]
10	Breeders and layer chicken	Characterized the lower respiratory microbiota in birds	16S (v1-v5) rRNA Amplicon Sequencing	Trachea and BAL	*Avibacterium, Pseudomonas,* and* Bordetella*	Several pathogens including *Myroides sp* *MY15*, *Collinsella aerofaciens, Bacteroides fragilis, Enterococcus cecorum,Kurthia zopfi*i, and *Kushneria sinocarnis sp* were detected	[5]
11	Broiler chickens	Evaluated the presence of *E. coli* in the respiratory system of healthy broilers	Plate culture	Trachea, lungs, and air sacs	*E. coli* bacterium detected in the respiratory microbiota, with a greater occurrence of this bacterium in the air sacs and lungs	*E. coli* strains isolated from the respiratory microbiota of healthy broilers do not present pathogenicity to the host	[133]
12	Layer chickens (white leghorn)	Compared invasive and non-invasive respiratory sampling techniques	16S V4 rRNA Amplicon Sequencing	nasal wash, trachea, and BAL	Nasal wash: *Lactobacillus, Ruminococcus, Lachnospiraceae, Rothia,* and *Vibrio* Trachea: *Lactobacillus, Vibrio, Halomonas, Pseudomonas,* and* Pseudoalteromonas* BAL: *Lactobacillus, Vibrio, Halomonas, Pseudomonas,* and *Pseudoalteromonas*	Non-invasive sampling (live-bird swabs) resulted in lower bacterial content compared with invasive sampling	[44]
13	Broiler chicken	Analyzed the influence of ambient air bacteria on the respiratory microbiota	16S rRNA Amplicon Sequencing	Trachea	*Gallibacterium,* Escherichia-shigella*, Romboutsia,* and *Enterococcus*	*E. coli* observed in the trachea are reported to be transient and facilitated by litter aeration by tumbling and aerosol formation	[53]
14	Broiler chicken	Explored the relationship between tracheal microbiota and inflammation under different levels of ammonia exposure	16S V3-V4 rRNA Amplicon Sequencing	Trachea	*Faecalibacterium, Lactobacillus, Escherichia-shigella, Ruminococcus, Acinetobacter, Lachnospiraceae,* and *Streptococcus*	High abundances of *Faecalibacterium*, *Blautia, Streptococcus, g__Ruminococcaceae_UCG-014, unclassified_f__Lachnospiraceae, Ruminococcus]_torques_group* in the trachea may result in the more release of IL-1β to damage the tracheal tissue	[13]
15	Layer chickens	Evaluated the effect of cyclic heat stress on the microbial diversity of the lungs	16S V3-V4 rRNA Amplicon Sequencing	BAL	*Janibacter, Brevibacillus, Serratia, Acinetobacter, Staphylococcus,* and *Lactobacillus*	Bacterial richness and bacterial diversity were reduced by heat stress Genus *Brevibacillus* was enriched in birds raised under thermoneutral conditions	[10]
16	Layer chickens and turkeys	Characterized the bacterial community of the chicken respiratory tract	16S V3-V4 rRNA Amplicon Sequencing	Trachea	Chicken: *Escherichia–Shigella, Enterococcus, Proteus, Macrococcus, Lactobacillus,* and *Staphylococcus* Turkey: *Enterococcus, Escherichia–Shigella, Lactobacillus, Proteus, Psychrobacter, Carnobacterium,* and *Streptococcus*	Bacterial composition of the respiratory tract of chickens is more diverse than that of turkeys	[9]
17	Layer chickens	Evaluated the effect of *Mycoplasma gallisepticum* infection on the diversity of the respiratory tract microbiota	16S V3-V4 rRNA Amplicon Sequencing	Trachea and BAL	*Lactobacillus, Burkholderia, Elstera, Acinetobacter,* and *Sphingomonas*	*Mycoplasma gallisepticum* infection altered the respiratory microbiota community	[157]
18	Broiler chickens	Evaluated the effect of danofloxacin in the treatment of *Mycoplasma gallisepticum* infection on chicken microbiota	16S rRNA Amplicon Sequencing	Lungs	*Bacteroidetes, Firmicutes,* and *Proteobacteria*	Firmicutes had a great decrease after challenge with *Mycoplasma gallisepticum* infection and an obvious recovery after treatment with danofloxacin	[28]
19	Layer chickens	Characterized the lung microbiome of laying hens across laying stages	16S V4 rRNA Amplicon Sequencing	Lungs	Bacteroidetes, Firmicutes, and Proteobacteria	Significantly higher Bacteroidetes and lower Firmicutes during peak lay compared with both early and late lay was observed	[158]

*S/N* Serial number, *BAL* Bronchoalveolar lavage, *DGGE* Denaturing gradient gel electrophoresis, *bTEFAP* Bacterial Tag-encoded *FLX* amplicon pyrosequencing, IL-1β Cytokine interleukin-1β. The table provides a comprehensive overview of studies investigating the respiratory microbiota in various poultry species, highlighting prevalent genera and their correlations with factors like bird performance and environmental stressors. Findings emphasize the significance of understanding the respiratory microbiome for optimizing poultry production and disease management strategies

In addition to the complexity of the poultry respiratory microbiota, inconsistency exists regarding the microbial diversity within various sites in the poultry respiratory tract. One theory is that microbial diversity decreases posteriorly along poultry respiratory sites, from the nasal cavity to the lungs, which has been referred to as the island ecological model in human studies [42, 43]. This proposition is grounded in an ecological framework, suggesting that the nasal cavities serve as a microbiota mainland (source), with the highest richness and evenness of microbial species, acting as a source of microbial population for other respiratory sites (sink) [44]. Several poultry studies [18, 24, 45] are in conformation with this proposition. Interestingly, Abundo et al. [44] reported an exception to this model as BAL samples recorded significantly higher microbial diversity compared to the trachea. This exception is herein referred to as the spatial-anatomical model. This model is based on the concept that the unique anatomical and physiological functions of each distinct respiratory site dictate the richness and evenness of its microbial residents. This would imply that constant mucociliary clearance occurring in the trachea contributes to its reduced microbial richness in comparison to LRT sites, where microbial deposition contributes to its higher microbial diversity [46]. Indeed, further studies are needed to clarify whether poultry respiratory sites exhibit a source-sink or unique microbial diversity trend.

### Influencing factors on poultry respiratory microbiota community composition and diversity

**Fig. 2:**
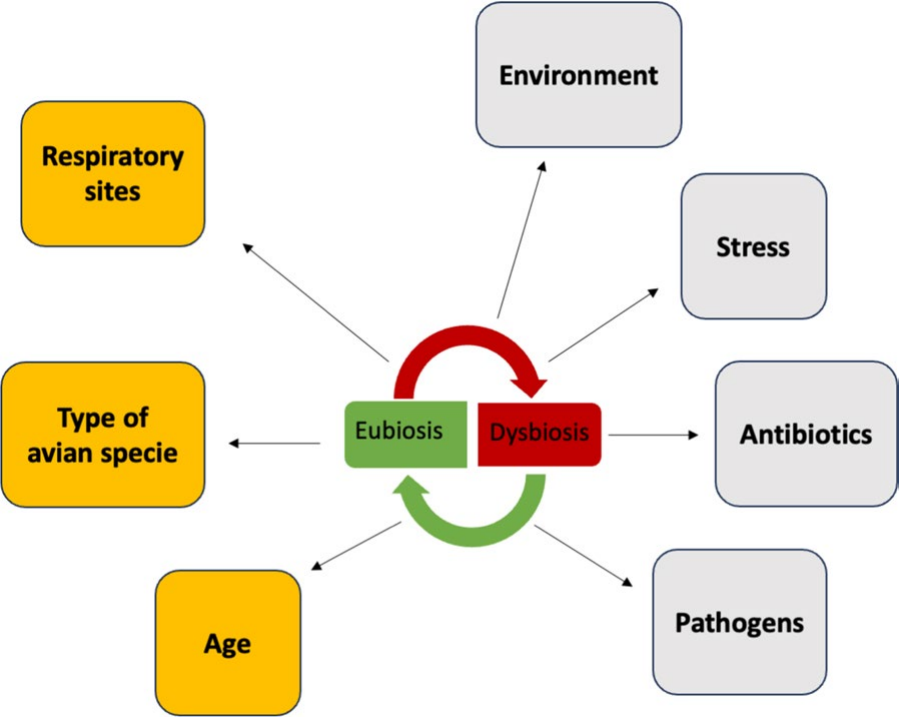
Factors shaping community structure and diversity of poultry respiratory microbiota. The interplay between intrinsic factors (highlighted in orange) and extrinsic factors (highlighted in grey) significantly influences the community structure and diversity of the poultry respiratory microbiota. The respiratory site itself, shaped by distinct physiological functions, determines the microbial residents, resulting in unique bacterial communities across various respiratory sites. Additionally, avian species type governs microbiota composition, notably between chickens and turkeys. Age is a critical factor, with microbial colonization increasing until an age-independent plateau is reached. However, this process is impacted by diverse stressors, environmental conditions, and management practices. Factors such as ammonia pollution, heat stress, antibiotic usage, and pathogen presence, especially viruses, can disrupt microbiota balance, potentially leading to dysbiosis. Understanding these intricate relationships is crucial for a comprehensive comprehension of avian respiratory microbiota dynamics

The composition and diversity of the respiratory microbiota in poultry are influenced by various factors. These include intrinsic host-related factors such as bird type, breed, age, and genetics, as well as several extrinsic factors. Extrinsic factors include production system, sampling sites and methods, sequencing platforms, and methodologies (choice of PCR primers, regions to be sequenced, number of PCR cycles, DNA extraction protocols), as well as nutrition, antibiotics use, disease, and environmental stressors such as heat and litter quality. The interplay of these factors contributes to the complexity of the poultry respiratory microbiota. A synthesis of available information on the most decisive determinants of the poultry respiratory microbiota (presented in Fig. 2) are described below:

### Intrinsic factors

As highlighted in our description of the spatial-anatomical model of microbial diversity in the respiratory tract, the distinct physiological and adaptive functions of each respiratory site determine its specific microbial residents. Hence, it is not surprising that the respiratory site under consideration is a factor that potentially influences microbiota composition and diversity. Several poultry studies have reported distinct bacterial communities across different respiratory sites [5, 24, 25, 34]. Sohail et al. [12] also reported distinct spatial bacterial ecology between the ceca and trachea using Polymerase chain reaction/denaturing gradient gel electrophoresis (PCR/DGGE) fingerprinting methodology. Tracheal samples were predominantly dominated by *Blautia*, *Clostridium*, *Eubacterium*, *Faecalibacterium*, *Heliobacterium*, *Ruminococcus*, *Oscillibacter*, and *Oscillospira* whereas cecal samples had *Aerococcus*, *Anaerococcus*, *Globicatella*, *Lactobacillus*, *Nosocomiicoccus* and *Staphylococcus* as the prevailing genera in their study. In conformation with results from Sohail et al. [12] study, Kallapura et al. [47] also reported a higher rate of *Salmonella* recovery in the trachea compared to the ceca. This disparity could possibly be because of the two mucosa milieus, the trachea is the easiest route for microbe entry and colonization. Additionally, longer feed retention time, and lower pH afford the ceca its unique bacterial composition and diversity [48]. Taylor et al. [18] provided unique perspective on the peculiarity of the poultry respiratory tract microbiota, as they report unique microbiota compositions of the avian trachea and nasal cavity. It is speculated that the avian trachea harbors more transient species compared to the nasal cavity. Similar observations have been reported in the human respiratory tract [6, 49].

Furthermore, the type of poultry bird is another factor that reportedly dictates the composition and diversity of the poultry respiratory tract microbiota. Although some bacterial taxa overlap occur, distinct differences in the URT microbiota (trachea) of chickens and turkeys have been reported [9]. Kursa et al. [9] further showed that the chicken trachea harbors significantly greater bacterial diversity compared to the turkey trachea. This difference in respiratory microbiota composition and diversity is speculated to be confounded by the age of the flocks, as the average age of chickens and turkeys from which samples were obtained in the study was 30 and 25 weeks, respectively [9].

The impact of age on the composition and diversity of the microbial communities in the avian respiratory tract has received less attention compared to the gut. While there have been several poultry studies demonstrating unique age-driven shifts in microbial composition across different intestinal sites [50–52], the role of age in the poultry respiratory microbiota remains relatively understudied. The first study to provide insight into the role of age on the poultry respiratory microbiota was conducted by Shabbir et al. [5], which characterized the lower respiratory microbiome of birds from different age groups (36, 40 and 55 weeks) from three different farms in the Punjab province of Pakistan. While the study reported unique differences in microbiota composition and diversity across the evaluated samples, it is difficult to attribute the observed effects solely to the age of the flocks. Multiple confounding variables from experimental design, including differences in sex, bird type (layers vs breeders), production system (open-house vs. free-range vs. controlled-house), and small sample size could have contributed to the observed outcome. The comparative study of Glendinning et al. [24] offered better perspective on the effect of age on the respiratory microbiota. The study reported different nasal microbiota composition from birds of different age groups, and it was found that two-day old and three-week-old birds recorded a high abundance of *Lactobacilli*. However, 30-month-old birds recorded a lower abundance of *Lactobacilli*, while *Jeotgalicoccus* and *Staphylococcus* were the most abundant genera in the 30-month-old birds. Additionally, microbial richness and diversity increased in buccal and nasal samples with increasing age, except for BAL samples. Another study by Ngunjiri et al. [25] reported a significant shift in nasal microbial communities in layers at 16–25 weeks of age compared to brooding (0–5 weeks of age) and grow-out (6–16 weeks of age) stages. This finding is also supported by the results from Kursa et al. [9] study, which showed increased numbers of unique Amplicon Sequence Variant (ASV) in tracheal samples as turkeys aged, suggesting increased microbial richness with increasing age. Interestingly, a similar observation was also noted for chickens in the same study.

While these findings help to justify that a direct linear relationship exists between bird age and microbial composition and diversity in the respiratory tract, this may not be the case. Our speculation is that during the first weeks of life, microbial colonization and diversity increases until an age-independent microbial stabilization plateau is reached. Several factors, including the environment, stressors, and nutrition could potentially contribute to when the microbial plateau is achieved. A good buttress of this hypothesis is the observed significant taxonomic successional difference in the microbiota of old and young layer chickens [25]. As old layers are more exposed to several physiological and management stressors, they undergo more microbial plasticity compared to younger hens, implying they take a longer time (age) to reach a microbial stabilization plateau. Furthermore, the study by Taylor et al. [18] suggested that there might be a respiratory-site specific effect of age on poultry respiratory microbiota. The study reported significant differences in species richness and evenness in the nasal cavity compared to the trachea. Anatomically, the nasal cavity's constant interaction with the external environment may explain the observed difference. Summarily, while age plays a significant role in shaping the poultry respiratory microbiota, other factors may also contribute to the observed microbial composition and diversity. Further studies are thus needed to provide more insight on the complete role of age on poultry respiratory microbiota.

### Extrinsic factors

It is common knowledge that a bird’s phenotype is largely influenced by its genotype and environmental influence. Hence it is not surprising that the environmental conditions of a bird affect its respiratory microbiota composition and diversity. For instance, the immediate environment of a poultry bird includes litter, feces, and moisture. All of which can potentially serve as a viable ground for pathogenic bacteria proliferation, depending on the stocking density, management conditions, and other factors. Recently Ivulic et al. [53] revealed that litter management could influence the respiratory microbiota of broiler chickens via an increase in atmospheric bacteria load. The authors conducted a longitudinal study to evaluate the effect of ambient air in a tunnel-ventilated broiler house on the tracheal microbiota. The tracheal microbiota was dominated by *Escherichia coli/Shigella* at week 3 of their study, which was correlated with litter aeration by tumbling. While litter aeration increases aerosols in the bird-rearing facility, it also prevents litter compaction brought on by moisture buildup from bird excrement, drinker leakage, and other factors [54]. Ivulic et al. [53] found that air tumbling as a management strategy reduced microbial diversity in the same period across the broiler rearing facilities in their study. High atmospheric bacterial load and temporal increase in ammonia levels in the barn facilitated by the litter aeration by tumbling are deemed responsible for the unique tracheal microbiota composition observed in their study. Poor barn ventilation, which results in high ammonia and dust concentrations, can be predisposing factors for pathogenic *E. coli*, such as Avian Pathogenic *Escherichia coli* (APEC) colonization of the bird respiratory tract [55]. In light of the implications of APEC, on bird health and welfare, it is imperative to closely examine innovative strategies for managing litter and barn environmental air. Furthermore, Ivulic et al. [53] reported a succession of tracheal microbiota from *Escherichia coli/Shigella* to *Gallibacterium sp*. at week 6, this reinforces the transient nature of *E. coli* observed at week 3. Additionally, given that microbiota dysbiosis often leads to inflammatory reactions [56, 57], it is plausible that the immuno-mucociliary clearance in the trachea contributed to the observed microbial succession.

As highlighted above, ammonia pollution in poultry barns may promote pathogenic microbial colonization in the respiratory tract, which in turn disrupts respiratory health [58, 59]. The avian trachea is thought to be affected by ammonia stimulation, resulting in a concurrent shift in the microbial composition. Zhou et al. [13] confirmed this in their study which evaluated the effects of varying levels (0–35 ppm) of ammonia on broiler chicken tracheal microbiota. Exposure to 15–35 ppm levels of ammonia disrupted microbial homeostasis in the trachea, as evidenced by reduced alpha and beta diversity. Increasing levels of ammonia exposure also increased the communities of *Blautia* and *Streptococcus.* Species from both genera have been linked to multiple respiratory pathologies and are capable of preventing the colonization of symbiotic bacteria [60, 61]. This result emphasizes the necessity of implementing innovative litter management and anti-pollution strategies.

Environmental stress, such as heat stress, is another factor that can induce a shift in respiratory microbiota. Oxidative stress, immunosuppression, inflammatory conditions, and microbial dysbiosis are a few of the negative impacts that heat stress can have on poultry [62, 63]. Relative to the intestine, the impact of heat stress (HS) on the respiratory microbiota is understudied. Several poultry studies [64–67] have recorded unique shift in gut microbiota in response to heat stress. In terms of the respiratory tract, Wang et al. [10] reported reduced bacterial richness and diversity in the lung microbiota of cyclic heat-stressed (30 °C, 10 weeks) layer-type pullets compared to their thermoneutral counterpart (24 °C). In terms of microbiota composition, significant reduction in members of the genus *Brevibacillus* was also recorded in the heat-stressed birds. This is a notable observation given that *Brevibacillus* species are often considered for probiotic use due to their ability to secrete antimicrobial peptides that can hinder the growth of pathogenic microorganisms [68]. In conformity with Wang et al. [10] result, Sohail et al. [12] also recorded reduced tracheal microbial diversity in broiler chickens subjected to chronic HS (35 ± 2 °C, 42 days). Additionally, their study provided insights into the limitations of popular methods utilized in microbiome research, particularly related to taxonomic resolution, such as PCR-DGGE and 16S rRNA throughput sequencing. Results obtained using PCR-DGGE showed that the impact of HS on the ceca was more pronounced compared to trachea. Moreover, dietary supplementation with mannooligosaccharides was found to restore tracheal microbial homeostasis in heat-stressed birds. In addition to identifying differences in microbial composition between HS and thermoneutral groups, the use of 16S rRNA high throughput sequencing provided distinct bacterial clades and relative abundances between treatment groups at a much lower taxonomic resolution. Taken together, these results demonstrate that HS induces respiratory microbiota community disruption and microbial diversity reduction. The extent of this microbial dysbiosis resulting from HS may vary based on factors such as the type, intensity, and duration of the stress [69]. Therefore, it is crucial to continue studying heat stress intervention strategies for the poultry industry, as the documented effect of reduced microbial diversity on decreased resistance to opportunistic infections has been well-established [70, 71].

Although yet to be fully explored, antibiotic use is another factor that can influence the poultry respiratory tract microbiota community structure. For over six decades, antibiotics have been used at sub-therapeutic doses to enhance the growth performance of poultry flocks [72]. However, their continued use has been linked to public health concerns related to antibiotic resistance and residues, leading to calls for their reduction and ultimate discontinuation by governments and the general public [73]. Several studies [71, 74, 75] have affirmed that antibiotics are capable of inducing changes in microbial structure in the poultry gut. This antibiotics-induced shift in microbial structure has also been associated with decreased adaptive immune responses in poultry, particularly in a dose-dependent manner [76–78]. Conversely, only one study [18] investigated the influence of antibiotics on the poultry respiratory microbiota. In this study, commercial turkey flocks treated with chlortetracycline recorded a lower abundance of *Mycoplasma* in the trachea. The abundance of *Mycoplasma* was negatively correlated with the average weight of birds in the same study, which is notable considering a link between gut microbiota composition and bird weight has previously been established [79]. Vancomycin-colistin induced gut microbiota dysbiosis with concomitant impairment of lung immunity has also been reported in mice [80]. Despite these findings, further studies are required to fully understand the role of antibiotics in dysbiosis of the poultry respiratory tract and its effects on immunity and growth performance.

Pathogens, especially viruses, have also been shown to cause respiratory microbiota disruption in humans [81–83]. This has recently been demonstrated in the poultry respiratory tract in a longitudinal study in which birds were challenged with low pathogenic avian influenza (LPAI) H5N2 virus [84]. Viral infection increased the total bacterial content in nasal microbiota samples; however, this increase had an inverse relationship with bacterial species richness. Samples retrieved from the nasal cavity also showed significant virus-induced change in beta diversity. Results from this study also suggest that virus-induced microbiota disruption is respiratory-site specific. Compared to the control treatment in this study, virus-induced microbial disruption across several taxa reduced linearly from the URT to the LRT. The number of microbial taxa disrupted by the LPAI H5N2 virus were 16, 5, and 1 across the nasal cavity, trachea, and lungs, respectively. Additionally, genera *Clostridium, Enterococcus, Escherichia-Shigella*, and *Pseudomonas* were positively correlated with viral titers in the nasal cavity, while genera *Enterococcus*, unclassified *Erysipelotrichaceae*, *Escherichia-Shigella, Staphylococcus*, and *Pseudomonas* were positively correlated with viral titers in trachea. Conversely, the *Lactobacillus* genus was negatively correlated with viral titers in the trachea. In conformation with our respiratory site-specific microbiota disruption hypothesis, Yildiz et al. [83] have also reported marginal effects of Influenza A virus infection on lung microbiota (LRT) in a murine model. Furthermore, considering that active virus replication occurred between days 5–7 post infection [83], another important finding from this study is that microbiota disruption may persist even after virus clearance. The reduction in bacterial species richness was still observed in samples retrieved from the nasal cavity even at 14 days post infection. Innate immune responses, particularly via cytokine production related pathways, is a possible mechanism for the virus-mediated microbiota dysbiosis. This has been pointed out in several in vivo mouse and poultry studies [71, 85–87]. Therefore, virus-induced dysbiosis could potentially support the proliferation of pathobionts and opportunistic infections in poultry flocks [88, 89]. It is yet to be seen if probiotic formulations can restore microbiota homeostasis in the poultry respiratory tract, following viral challenge.

Despite the very limited number of studies that have explored the effects of various intrinsic and extrinsic factors on the poultry respiratory microbiota, it is important to state that in the absence of well-designed a priori experiments, it is difficult to determine which of these factors exert the greatest effects. Much of the available data in the literature are confounded by several variables in their experimental design, hence the interpretation of these results needs to be treated with caution. For instance, multivariate regression models revealed that experimental factors including bird type, age, body site, and flock in the study by Taylor et al. [18] were only able to explain about 54% of the total sample variance. This suggests the existence of additional sources of microbial heterogeneity beyond those accounted for. The information provided here on select intrinsic and extrinsic factors that potentially influence the respiratory microbiota would be valuable for the design and execution of poultry microbiota studies with reproducible outcomes.

### Role of poultry respiratory microbiota in pathobiology and immune responses

The avian mucosal surfaces are occupied by an abundant number of microbial communities that are in constant interaction with the host, other members of the community and the environment. A healthy balance of this tripartite relationship, especially in terms of compositional stability and diversity, ensures a healthy microbiota in humans [90]. Any compositional or functional alteration of this relationship is referred to as microbial dysbiosis, which is generally characterized by increased abundance of pathobionts [91]. Microbial dysbiotic conditions are implicated in several pathological conditions in avian species, including pathogen colonization, excessive inflammation, dysregulated immune responses, and severe disease conditions [33, 92]. The potential of the respiratory microbiota to influence viral infections, vaccine responses, secondary bacterial infections, and disease severity has also been reported [12, 93]. Consequently, the role of resident microbiota in the poultry respiratory tract on the host immune system and disease outcomes continues to be the subject of recent research [13, 94]. Information from studies of this nature could offer a better understanding of economically important avian diseases and zoonoses.

Economically important avian respiratory diseases that are microbiota-exacerbated or linked to bacterial etiologies include but are not limited to *Mycoplasma* infection, infectious coryza, Colibacillosis and Avian Influenza [10, 28, 71, 95–99] (Table 2). Under homeostatic conditions in the respiratory tract, commensal microbiota contribute to host immunity by inhibiting pathogen persistence and transmission via several means, including competitive exclusion, bacteriostatic activities, biofilm formation, quorum sensing, and others [100]. Conversely, under dysbiotic conditions, potential respiratory pathogens include *Escherichia coli*, *Ornithobacterium rhinotracheale, Mycoplasma synoviae*, *Mycoplasma gallisepticum*, *Gallibacterium anatis*, *Avibacterium* species*,* and *Staphylococcus aureus* [9, 33, 101]. The relative abundance of some of these microbial species has been found to be consistent between both healthy and infected poultry respiratory tracts [9], suggesting that under specific conditions like stress and viral infections, pathobionts may transition into pathogens. The specific roles of microbiota in select important poultry diseases are subsequently reviewed below.

**Table 2:** Distinct bacterial taxa linked to economically important respiratory diseases in poultry

Serial number	Poultry diseases	Type of poultry bird	Associated bacterial taxa		References
			Increase (+)	Decrease (−)	
1	Avian Influenza	Layer chickens	*Proteobacteria, Vampirovibrio*, Ruminococcus, Alistipes, *Enterobacteriaceae, Escherichia, Clostridium,* and *Veillonella* Pseudomonadales	*Lachnospiracea, Ruminocacaceae, Enterococcus, Lactobacillus, Streptococcus,*	[71, 97, 98]
		Waterfowl	*Micrococcaceae*	*Streptococcus, and Veillonella,*	[95]
2	Mycoplasma	Layer-type pullets and broiler chickens	*Bacteroides, Enterococcus, Prevotella, Pseudomonas, Acinetobacter,* and *Serratia marcescens*	*Staphylococcus, Lactobacillus, Weissella,* and* Butyricicoccus*	[10, 28]
3	Colibacillosis	All types of poultry bird	*Avian Pathogenic Escherichia coli*	–	[96]
4	Infectious coryza	Layer chickens	*Staphylococcus chromogenes, Escherichia coli* and* Pasteurella multocida*	–	[99]

Table 2 outlines bacterial taxa associated with significant respiratory diseases in poultry, indicating specific increases or decreases in abundance during infections like Avian Influenza, Mycoplasma, Colibacillosis, and Infectious Coryza across various poultry types. These findings emphasize the importance of understanding bacterial dynamics in respiratory diseases for effective disease management in poultry production

### Avian influenza viruses

Avian influenza is a global zoonotic disease that continues to threaten the economic efficiency of the poultry industry. Taxonomically, Avian Influenza Viruses (AIV) belong to the order *Mononegavirales,* family *Orthomyxoviridae,* and genus *Influenza A* [102]. The highly pathogenic subtypes of these viruses are the causal factors for respiratory infections associated with high morbidity and mortality rates in poultry [103]. The oropharyngeal cavity is the main and initial point of initial AIV replication, which is followed by replication in other sites including the gut [104, 105]. The natural host of AIVs are waterfowls [106]. On the basis of pathogenicity, there are two major AIV pathotypes, high pathogenicity avian influenza (HPAI) viruses (e.g., H5N1) and low pathogenicity avian influenza (LPAI) viruses (e.g., H9N2) [107]. While LPAI are tropic to the gastrointestinal tract in poultry, HPAI have tropism for the respiratory tract [108]. However, recent studies have also reported the presence of LPAI viruses in other tissues including brain, cardiac, splenic, hepatic, and renal tissues in chickens [108]. Virus pathotypes also influence their virulence factors, as HPAI viruses can cause up to 75% mortality in infected chickens, whereas LPAI is less virulent [109]. Nonetheless, the LPAI H9N2 subtype has been reported to attain a panzootic state in poultry [110]. Other than pathotypes, the age and immune status of the host have also been shown to affect morbidity and mortality [107]. Signs of AIV infection are also influenced by pathotype and can range from mild lethargy, diarrhea, mild respiratory distress, and reduced egg production in LPAI virus infected hosts, to more severe clinical outcomes like coughing, sneezing, cyanosis of combs and wattle, hemorrhages on the shank, bleeding from the nares, incoordination, and death, which is observable in hosts with HPAI infection [104, 111].

Irrespective of the pathotype, commensal microbiota contributes to inducing immune responses against AIVs [112]. Contrastingly, microbiota dysbiosis, especially in the gastrointestinal tract, has been shown to increase the severity of AIV infection [113]. A good account is the study of Zhao et al. [114], which reported a modulation in the dominance of bacterial genera such as *Lactobacillus* and *Aeromona*s in the gut and fecal microbiota of HPAI H5N1 infected Whooper swans. Notably, this shift in microbiota homeostasis was associated with increased AIV transmission. Several avian models have confirmed the impact of compositional changes in the microbiota on immune responses against AIV in both the gut and trachea [71, 87, 111, 115]. By depleting the microbiota using antibiotics, Figueroa et al. [115] were able to confirm an increase in H5N9 viral replication and a reduction in antiviral immune response in infected ducks. This was evidenced by a reduction of interferon (IFN)-α–induced gene expression and over 100-fold increase in viral cloacal shedding in antibiotic-treated ducks at 3 days post infection. Intestinal tight junction protein (Mucin 2) was also significantly reduced in H5N9-infected ducks treated with antibiotics in their study, suggesting that microbiota dysbiosis could also impair gut integrity during AIV infection. The observation that microbiota depletion influences viral replication and antiviral immune responses has been well substantiated in several murine and avian models [71, 87, 116, 117]. Rowe et al. [118] also showed that common members of the human nasopharyngeal microbiome, such as *S. pneumoniae*, *M. catarrhalis*, and *H. influenzae*, are associated with increased AIV replication in a ferret model.

In addition to modifying the structural composition of the microbiota, another way in which AIV infection influences antiviral immune response in the host is by influencing microbiota diversity. There are conflicting reports on the effect of AIV infection on microbiota diversity in the literature. Kaul et al. [97] reported significantly higher microbial diversity (Beta diversity) in AIV-infected ferrets compared to uninfected ferrets. These microbial perturbations are facilitated by host antiviral responses, such as the induction of interferon [97]. In contrast, Chrzastek et al. [111] reported reduction in microbial richness and phylogenetic diversity in the colon microbiota of H9N2 AIV-infected birds. Similar reduction in species richness have been reported in the fecal microbiota of H5N1 AIV-infected swans [114]. In spite of this inconsistency related to the effect of AIV infection on microbial diversity in the literature, a simplistic definition of “low” or “high” levels of diversity and their relation to host antiviral immune responses may not be entirely correct. A closer look at the specific colonizers of the microbial community and the ecological niche occupied may afford better insight. For example, microbiota dominated by Proteobacteria, *Vampirovibrio*, *Ruminococcus*, *Alistipes*, *Enterobacteriaceae*, *Escherichia*, *Clostridium*, and *Veillonella* may signify specie richness. However, these taxa have been associated with increased AIV replication [71, 98]. It is also possible that the activities of this taxa differ depending on the ecological niche occupied. Also, they may increase AIV virulence in respiratory sites compared to the gut. Nonetheless, being the main site of infection, the effect of AIV infection on the respiratory microbiome requires more attention. Conversely, certain microbial species have demonstrated positive impacts on adaptive immune responses and antiviral responses. For instance, Alqazlan et al. [119] showcased the ability of specific *Lactobacillus* strains (such as *L. salivarius, L. johnsonii*, and *L. reuteri)* to induce immunostimulatory and antiviral responses against H9N2 AIV. These responses encompass the activation of interferon-stimulated gene (viperin) and the robust expression of cytokines in cells of the ceca tonsils, including T-helper (Th)1 type cytokines (interleukin [IL]-2, IL-12, and IFN-γ), pro-inflammatory cytokines (IL-1β and IL-6), and an immunoregulatory cytokine (IL-10). Moreover, a combination of *Lactobacillus* species, comprising *Lactobacillus salivarius, L. johnsonii, L. reuteri, L. crispatus,* and *L. gasseri*, has also been shown to enhance both cell- and antibody-mediated immune responses against H9N2 AIV [78]. This beneficial effect is particularly pronounced when co-administered with synthetic CpG oligodeoxynucleotides (ODN) 2007.

### Avian mycoplasmosis

Avian *Mycoplasmosis* is a chronic infectious respiratory disease that affects poultry of all ages. The economic consequence of this disease includes poor hatchability, reduced egg production, poor weight gain and egg production [120]. The etiological agent of this disease is the pathogenic bacterium, *Mycoplasma gallisepticum* (MG), which is commonly isolated on most poultry farms. This pathogen can be spread both vertically and horizontally and can cause severe inflammatory responses and immune dysregulation in birds [120, 121]. Current management tools available to curb MG infection on farms include sourcing birds from breeding stock that are certified as healthy, implementing rigorous biosecurity practices, administering vaccinations, and delivering antimicrobials to infected birds [122, 123]. Although the use of antibiotics like fluoroquinolones and macrolides is a popular approach, there is a growing concern related to the development of antimicrobial resistance in this pathogen [124, 125].

By combining both 16S rRNA sequencing methods and an antibiotic-induced microbiota depletion protocol, Wang et al. [10] were able to provide insight into the role of the respiratory microbiota on MG infection in layer chickens. The findings of this study revealed that MG infection induces a distinctive shift in the microbiota community. Specifically, there was a significant decrease in the relative abundance of bacteria such as *Staphylococcus*, *Lactobacillus, Weissella*, and *Butyricicoccus* in their study. *Staphylococcus* and *Lactobacillus* genera have been identified as microbiota associated with optimal respiratory health [126]. On the other hand, the relative abundance of potential pathogens that include Bacteroides, *Serratia, Enterococcus, Prevotella, Pseudomonas* and *Acinetobacter* was increased by MG infection [10]. Furthermore, to directly associate specific bacteria with MG infection, nine Gram-negative bacterial species (*Serratia marcescens, Bacteroides ovatus, Parabacteroides distasonis, Prevotella copri, Subdoligranulum variabile, Bacteroides fragilis, Acinetobacter calcoaceticus, Proteus mirabilis* and *Pseudomonas aeruginosa*) were isolated from chicken BAL samples and cultured. Results showed that of all isolated bacteria, only *Serratia marcescens* significantly increased MG colonization in the respiratory tract when intranasally delivered to chickens [10]. Similar results of microbial disruption following MG infection have also been reported in the chicken gut [127].

In addition to disrupting the respiratory microbiota community structure, MG infection also affects microbial diversity. Shannon and Chao diversity indices were both significantly reduced by MG infection compared to the control treatment in a study by Wang and colleagues [10]. This decrease in diversity of the respiratory tract following MG infection is consistent with further works from the same group [28]. The disruption of microbial homeostasis in the respiratory tract recorded in these studies triggered a concomitant immunological response and tissue damage. MG-infected chickens with disrupted microbiota exhibited increased levels of levels of pro-inflammatory cytokines (IL-1β, IL-8 and IL-6) in the lungs and severe lung tissue damage [10]. Despite the immunological challenge that microbiota dysbiosis creates, microbiota manipulation could also potentially afford a solution to this challenge. Some studies suggest that the use of Gram-positive bacteria like *Lactobacillus* and *Staphylococcus* may facilitate protection against respiratory infection [126, 128]. This use of probiotics or direct-fed microbials requires more research in order to ensure therapeutic effectiveness in poultry.

### Colibacillosis

Colibacillosis is another poultry disease that causes significant economic losses, especially in terms of morbidity, mortality, stunted growth performance, and carcass condemnation [122]. It is an extra-intestinal disease with respiratory origin that triggers *colisepticemia* and systemic infections [129, 130]. APEC is the causative organism for this disease in poultry. APEC colonization begins in the trachea and air sacs, where it can induce airsaculitis before progressing to the lungs, liver, and pericardium [96, 131].

Although the influence of the microbiota on this disease has been documented in the gastrointestinal tract [130, 132], a few studies have also confirmed the presence of *Escherichia coli* in the respiratory microbiota of poultry [33, 133, 134]. However, the isolated *Escherichia coli* strains from these studies were confirmed to be non-pathogenic to the host [133, 134]. This observation is not surprising as the severity of APEC infection is dependent on strain pathogenicity and gene virulence [135, 136]. In the chicken gut, microbiota depletion has been shown to induce increased susceptibility to APEC infection [130]. Similarly, APEC infection induces gut microbiota dysbiosis and increased microbial diversity in chickens [137]. Nonetheless, considering that the respiratory tract is the site of initial APEC colonization, it is evident that greater attention needs to be paid to the impact of APEC on respiratory microbiota homeostasis in the future.

In summary, it is clear from the above information that the microbiota plays a significant role in respiratory dysbiosis and disease conditions in poultry. However, there are only a limited number of studies focusing on the respiratory microbiota, thus much more research is needed to understand the underlying mechanisms of microbiota perturbations and community shifts. Additionally, while various bacterial species have been linked to multiple infectious disease pathologies (as shown in Table 2), it is crucial to recognize that correlation does not always imply causation. Therefore, it is necessary to use bacteria-specific culture methods to isolate and examine the function of these microbial species more closely.

### Challenges in studying poultry respiratory microbiota

With the scarcity of information in the area of the chicken respiratory microbiota, our understanding in this field is still evolving. Notably, a myriad of confounding variables related to sampling methodology exist. Hence, there is a need to establish standardized methodologies regarding these confounding variables in the field in order to permit inter- and intra-study comparison of results.

Potential confounding variables include variations in sample collection methods (invasive vs. non-invasive sampling), storage and processing protocols, DNA extraction kits and procedures, and the use of different bioinformatic approaches. Based on the spatial-anatomical microbial diversity hypothesis (described in Sect. 2), each respiratory site in poultry is thought to host a distinct set of microbial species with varying levels of diversity. Therefore, it is challenging to determine which respiratory site accurately represents the core poultry respiratory microbiota. In a study comparing multiple respiratory site samples (choanal swabs, nasal wash, tracheal wash, and lower respiratory lavage), Abundo et al. [44] were able to confirm that tracheal washes and lower respiratory lavage samples afforded the most consistent and reproducible results regarding respiratory microbiota community compositions compared to other respiratory sites. The study also found that bacterial community compositions obtained from nasal washes were inconsistent between experiments. Considering that the nares are in constant interaction with the bird’s external environment, they may thus be predisposed to environmental microbiota contamination. For this reason, Abundo et al. [44] recommends the inclusion of an environmental control group in studies sampling the nasal microbiota. Preliminary data from our laboratory (unpublished data) agree with the findings of Abundo et al. [44]; tracheal and BAL samples were found to be the most consistent sites of URT and LRT regarding bacterial community composition consistency, respectively. Our group has also previously identified the optimal sample size (*n* = 5 chicken samples) suitable for poultry microbiota research [138].

Another important consideration is the choice of using invasive or non-invasive sampling techniques. While invasive sampling techniques yield high microbial biomass from euthanized birds, continuous sampling of the same bird is impossible, negating the feasibility of a longitudinal study [9, 25, 33]. In contrast, non-invasive techniques (live bird swabs of various respiratory sites) tend to yield lower bacteria density and are more prone to contamination due to the multiple sample processing steps [44, 139]. In addition to pelleting by centrifugation and other DNA extraction steps common to both invasive and non-invasive samples, live bird swabs (non-invasive samples) involve additional processing steps such as immersing the swabs in a transport/storage media, vortexing to facilitate the elution of organic material, including microbes, and an increased requirement for a higher DNA lysis buffer in the DNA extraction process [44].

In addition to sampling methodology, Abundo et al. [45] showed that the choice of DNA kit could also influence results of respiratory microbiota density and diversity. In order to ensure validity and reproducibility of results, it is recommended that only kits specifically certified for microbiome sample extraction are utilized. Adherence to “The Minimum Information for Publication of Quantitative Real-Time PCR Experiments (MIQE) guidelines” and adapting the “STORMS checklist” [140] are other ways of ensuring data reliability across studies. In addition to these guidelines, the field of animal microbiome research is in need of comprehensive and standardized guidelines to facilitate research reproducibility and consistency. Furthermore, Abundo, [141] makes a valid case for the inclusion of positive controls, i.e., microbial communities with well-defined bacterial composition in microbiome studies. The inclusion of reference standards, whether positive or negative, could help mitigate variations due to sample collection, processing, and sequencing biases.

### Gut-respiratory microbiota cross talk?

Primordially, the gut and the lungs differentiate from the same stem cells [142]. Additionally, microbiota and mucus are essential components of both the gut and respiratory systems, and their interactions with mucosal membranes elicit diverse effects on host health [143–145]. The bi-directional communication facilitated by neurochemicals, hormones, immune system cells, and microbiota metabolites between the gut and the respiratory system is now termed the gut-lung axis communication [146, 147]. Gut microbiota dysbiosis affects respiratory sites via mucosal immunostimulation, while respiratory dysbiosis also is understood to influence gut functions via immune regulation [148, 149]. The gut-lung axis communication has been implicated in several human diseases, including inflammatory bowel disease, irritable bowel syndrome, Colitis, and Crohn’s disease [142, 150].

In poultry, evidence of gut-respiratory microbiota cross talk is emerging. In a study involving the microbial evaluation of the URT and gut of 181 layer chickens, Ngunjiri et al. [25] discovered up to 29% of analyzed operational taxonomic unit was shared between these sites. Both sites were dominated by microbes in the order *Lactobacillales, Clostridiales*, and *Enterobacterales*. The study reported significant compositional similarity between the URT and the ileum specifically. Possible rationale for these microbial interactions across both sites may include aerosolization of fecal bacteria, gastroesophageal reflux, or systemic circulation of gut-derived microbial metabolites [25, 151]. Similar microbial overlap has been reported in turkeys and broiler chickens [18, 34]. While Johnson et al. [34] showed that the tracheal microbiota slightly mirrors that of the gut in broiler chickens, Taylor et al. [18] speculated that bacteria in the gut migrate to the respiratory tracts via aerosolization. To further validate this cross talk between the gastrointestinal tract and the respiratory system, respiratory microbiota dysbiosis has also been shown to be capable of potentially inducing gut microbiota dysbiosis. For example, inhaled ammonia was found to induce tracheal injury and tracheal microbiota dysbiosis in ammonia-challenged broiler chickens [152]. This ammonia exposure subsequently triggered gut microbiota dysbiosis via direct and indirect mechanisms including gut leakage and Toll-like receptor signaling pathways [152]. In addition to ammonia exposure, other stressors including heat stress have been reported to perturb the microbial community structure and diversity in the intestinal and respiratory sites concurrently [10]. These results emphasize the potential role of microbial cross talk in poultry susceptibility and resilience to environmental stressors, and the need for additional studies to provide more insights on the microbial relationship between these sites.

### Potential implications for poultry health

The potential for the gut microbiome to influence microbial compositions and functions in other tissues, particularly respiratory sites via the gut-lung axis, presents the opportunity to develop therapeutic interventions in poultry for the gastrointestinal tract microbiota that extend beyond the gut [153]. Although the mechanism of action is yet to be fully delineated, several studies have affirmed the contribution of gut microbiota in immune responses to respiratory infections in poultry.

Using an antibiotic-mediated microbiota-depletion protocol, Peng et al. [130] showed that gut microbiota depletion increased the severity of colibacillosis induced by intratracheal inoculation of APEC in chickens. The results showed increased production of proinflammatory cytokines IL-1β and IL-6, both at transcript and protein levels, in addition to significant pathological damage to the lungs in gut microbiota-depleted chickens compared to the control group. Conversely, acetate, a microbiota metabolite, was found to inhibit lung pathological injury, bacterial load, and inflammatory cytokine production in chickens [130]. Gut microbiota-derived acetate has also been found effective against severe respiratory syncytial virus and AIV infection in mice [154, 155]. In conformation with this result, Yitbarek, et al. [71, 87] utilized antibiotic-mediated microbiota-depletion protocols to demonstrate that the commensal microbiota in the gut play a critical role in the infection and replication of H9N2 AIV in chickens. In both studies, microbiota depletion in the gut resulted in higher virus shedding compared to chickens with intact microbiota. Additionally, gut microbiota depletion triggered downregulation of type-I IFN expression in the gut and respiratory sites [71, 87]. Similarly, antibiotic-treated ducks demonstrated increased shedding of H5N9 HPAI virus and reduced antiviral immune responses in the gut [115]. The role of the gut microbiota in influencing immune responses in distant organs, such as the respiratory tract, has also been supported by numerous studies involving germ-free or antibiotic-depleted mice [116, 150, 154, 156].

The findings from these studies and models clearly demonstrate that depletion of the gut microbiota compromises immune responses against respiratory infections. Metagenomic sequencing has thus far provided a good understanding of commensal and pathogenic bacterial species associated with the reported immunosuppression. Nevertheless, there is a need for broader exploration of other microbial residents in the gut and respiratory tract, including fungi, parasites, and viruses, and their association with immune responses in poultry. Adopting a metabolomics approach would offer a better understanding of the roles and metabolic pathways associated with these microbes, going beyond a simple microbial census. This approach could facilitate the development and validation of important therapeutic metabolites and biomarkers for economically important poultry diseases.

## Conclusions

Although the available literature on poultry respiratory microbiota is still limited, it has contributed significantly to our understanding of the microbiota residing in the poultry respiratory tract. This knowledge has shed light on the role of the respiratory microbiota in poultry health and productivity, as well as the factors that affect the community structure. However, it is crucial to acknowledge that further well-designed studies are necessary to provide substantial insights into multiple essential areas. Firstly, respiratory tract microbial colonization requires attention. While it is hypothesized that colonization is dependent on maternal factors, the influence of the environment, particularly the hatchery environment, cannot be underestimated. Further studies are required to establish congruency in this regard.

Secondly, a clear direction on the state of microbial diversity across the poultry respiratory tract sites demands urgent attention. In addition to the island ecological model, which suggests that the nasal cavities harbor the most diverse microbiota among all respiratory sites, we have also highlighted a spatial-anatomical model based on available evidence. This model suggests the presence of distinct microbial diversity, independent of any specific respiratory site. Consequently, studies that consider the spatial uniqueness and temporal dynamics of the poultry respiratory tract microbiota, along with their impact on microbial diversity, will be highly beneficial.

It is evident that the balance between eubiosis and dysbiosis of the poultry respiratory microbiota plays a role in determining the resistance or susceptibility to respiratory infections. This can occur through immune-mediated responses or microbial metabolite signaling. For instance, increased abundances of *Proteobacteria, Pseudomonas*, Avian Pathogenic *Escherichia coli*, and *Staphylococcus chromogenes* have been associated with various respiratory infections. However, while poultry respiratory microbiota may contribute to increased susceptibility to respiratory infections, they might also serve as therapeutic remedies for such infections. Consequently, there is a need for comprehensive studies that employ both culture-dependent and metagenomic approaches to investigate the probiotic potential of the resident microbiota in poultry respiratory sites. The gut-respiratory microbiome cross talk highlighted here could potentially open up new therapeutic opportunities for managing poultry respiratory infections. Conclusively, although a considerable amount of research has been dedicated to studying bacterial residents in the poultry respiratory tract, equal attention, if not more, should be given to other community members in the poultry respiratory microbiota, including fungi, archaea, parasites, and viruses. A combination of metatranscriptomics, metaproteomics, and metabolomics approaches would facilitate a better understanding of their community structure, interactions with bacteria, and their role in poultry respiratory pathology.

## Data Availability

Not applicable.

## Abbreviations

AIV: Avian influenza viruses
APEC: Avian pathogenic *Escherichia coli*
ASV: Unique amplicon sequence variant
BAL: Broncho-alveolar lavage
CpG ODN: Oligodeoxynucleotides
HPAI: High pathogenicity avian influenza
HS: Heat stress
IFN: Interferon
IL: Interleukin
LPAI: Low pathogenic avian influenza
LRT: Lower respiratory tract
MG: *Mycoplasma gallisepticum*
MIQE: Minimum information for publication of quantitative real-time PCR experiments
PCR/DGGE: Polymerase chain reaction/denaturing gradient gel electrophoresis
Th: T-helper
URT: Upper respiratory tract
